# Competence in Spiritual and Emotional Care: Learning Outcomes for the Evaluation of Nursing Students

**DOI:** 10.3390/healthcare10102062

**Published:** 2022-10-17

**Authors:** Juan Antonio Sarrión-Bravo, Alexandra González-Aguña, Ricardo Abengózar-Muela, Alina Renghea, Marta Fernández-Batalla, José María Santamaría-García, Roger Ruiz-Moral

**Affiliations:** 1 Faculty of Health Sciences and Faculty of Medicine, Francisco de Vitoria University, Majadahonda, 28223 Madrid, Spain; 2 Healthcare Management of Primary Care, Community of Madrid Health Service (SERMAS), 28880 Madrid, Spain; 3 Research Group MISKC, Department of Computer Science, University of Alcala, Polytechnic Building, Alcalá de Henares, 28805 Madrid, Spain; 4 Henares University Hospital, Community of Madrid Health Service (SERMAS), 28822 Madrid, Spain; 5 Torres de la Alameda Health Centre, Community of Madrid Health Service (SERMAS), 28813 Madrid, Spain; 6 Meco Health Centre, Community of Madrid Health Service (SERMAS), 28880 Madrid, Spain

**Keywords:** clinical competence, competency, education, emotions, nurses, nursing students, spirituality

## Abstract

Spiritual and emotional care is an important part of the person, especially in situations such as changes in health or a community coping with a pandemic. However, nurses report scarce university training in this area of care. The aim of the study is to define a catalogue of learning outcomes for spiritual and emotional care for undergraduate nurses. The design used a mixed method for the development and validation of learning outcomes. The first phase designs the catalogue of learning outcomes through a coordinating group and uses a bibliographic search and nursing legislation. The second phase validates the proposal through a group of experts, with a questionnaire using the modified Delphi technique in two rounds. The initial proposal was 75 learning outcomes, of which 17 were eliminated, 36 changed their wording and the experts proposed 7 new ones. The experts validated 65 learning outcomes: 14 for Assessment and diagnosis; 5 for Planning; 17 for Intervention; 4 for Evaluation and quality; 8 for Communication and interpersonal relationship and 17 for Knowledge and intrapersonal development. In conclusion, the academic curriculum can include these learning outcomes to help undergraduate nurses in the process of acquiring knowledge, skills and attitudes in spiritual and emotional care.

## 1. Introduction

The Nursing Now movement emerged in 2018, and later the World Health Organisation (WHO) declared 2020 the International Year of the Nurse and Midwife. Nurses accepted the challenge of demonstrating their added value to society, their importance in health systems and their competence to lead decision-making [1,2,3].

For this leadership, nursing training programmes include a holistic perspective, where the centre of health care is the person in their environment. The nurse accompanies the person throughout life regardless of the circumstance of health or illness [4,5]. Nursing attends to physical health, but also psychological and spiritual health, both of the person and of families and communities [6]. Furthermore, the Code of Ethics of the International Council of Nursing affirms that nurses provide care by promoting an environment of respect for human rights, values, customs, religious and spiritual beliefs [7].

In this sense, at the end of December 2019, when nursing was going to begin its International Year to make care visible, the coronavirus disease, COVID-19, emerged in China, which resulted in millions of infections, hospitalisations and deaths all over the planet [8,9,10]. Nurses proved to be the backbone of health systems and to have the ability to adapt to any situation or emergency [1]. However, this crisis not only had an impact on physical health, but also had an impact on social, economic and, in parallel, mental health. The United Nations Organisation warned that the COVID-19 pandemic would increase the number and severity of mental health problems [11].

The entire population faced the risk of infection, periods of lockdown and a global crisis that made us reflect on present and future life. This reflection led to a situation of uncertainty where people reconsidered their lifestyle, culture, values and beliefs. The way in which an individual handles this situation can lead to stressful situations with negative effects on their health. Specifically, in Spain, 46% of the population experienced an increase in their psychological discomfort due to lockdown and diagnoses of depression, anxiety or suicide increased [11,12,13]. In this regard, health professionals received special attention from the beginning of the pandemic because they were a group on the front line of the health crisis; additionally, their health situation had repercussions on ensuring the continued human resources of the health services [12,13,14,15].

In short, the health problem, COVID-19, had an impact on multiple factors that characterise a culture: social, political, economic, educational or technological. Madeleine Leininger focused her theory on care (nursing centre) and culture. The Sunrise Model details the network of factors of the social structure that characterise a culture and that influence the individual and collective vision of the world. Some of those factors influence the conceptualisation of health and disease, and, consequently, decision-making and acts of care [4,16]. In this sense, Leininger defined culturally congruent care as knowledge-based acts and decisions for care that match the values, beliefs, and lifestyles of the individual or group in order to provide meaningful care that achieves progress to health and wellness [4,16,17]. This competence is a continuous process that allows for the formulation of appropriate care diagnoses and, therefore, for the application of appropriate care plans for each person [18,19]. This culturally congruent care framework is limited in this study to the ideas of spirituality and emotion, which have been called for this research as intimate care.

Spirituality is a subjective, dynamic and interactive process that can have a positive or negative impact on people’s health and, in the opposite direction, any change in health causes critical situations which need to be coped with by spirituality [20,21,22,23]. Spirituality includes religiosity, but it is not synonymous with it, because it includes many more aspects [6]. Spiritual care is “*an intuitive, interpersonal, altruistic, and integrative expression that is contingent on the nurse’s awareness of the transcendent dimension of life but that reflects the patient’s reality*” [24]. Spiritual care is a part of holistic nursing that concerns well-being and the perception of quality of care [25,26]. Giske conducted a review of the literature where she identified four essential areas for learning spiritual care, which are: “*(1) the importance of learning in real-life situations with repeated exposure to patients in diverse placements; (2) use of pedagogical methods that assist students to understand, work with and reflect on patients’ spirituality; (3) to be aware of and overcome conditions prohibiting spiritual care learning; and (4) to see spiritual care learning in connection with how students are prepared and how they are followed up after clinical studies*” [27]. Nardi and Rooda defined five dimensions of nursing practice based on spirituality. These dimensions of clinical practice are “*1: Valuing and supporting others, 2: Use of spirituality-based nursing process, 3: Use of the metaphysical self, 4: Individual spirituality-based action, 5: Spirituality-based outcomes*” [28]. Furthermore, Williams et al. evaluated the use of the FICA tool in the perception of spirituality of nursing students and the provision of spiritual care, which makes visible the needs in this area [29].

On the other hand, emotion is an alteration or change that occurs in response to an external or internal stimulus and that involves cognition, interpretation, physiological changes and a tendency to initiate motor actions and expression. Emotions are present in the relationships between professionals and patients during the care process and must be understood in order to approach the person’s meaning. In addition, the professionals must be aware of their own emotions and their impact on nursing care [30]. Thus, emotional competence is a combination of skills to develop in a cultural context through social experiences [31,32].

Spirituality and emotions are linked because spirituality is visible in the expressions and practices of care, and, furthermore, people externalize their internal responses through emotions. However, the nurses reported scant training in spiritual care, with an insufficient emphasis during their period as students [6,33].

Competence in care for both areas can be learned, as part of culturally consistent care, so university training programmes must include learning outcomes for undergraduates that make explicit these areas gradual acquisition [19]. Competence is defined as qualification or training, as a person’s level of experience and their degree of preparation. These competencies are complex acts that regulate the efficient combination of cognitive, affective and psychomotor aspects, such as knowledge, critical thinking, communication, attitudes and skills to respond to clinical situations [34,35]. In this sense, competencies will lead to the development of learning outcomes. Learning outcomes are “*verifiable statements of what a student should know, understand and be able to do*” [36] at the end of a course or when they have completed a programme [36].

Students must demonstrate the acquisition of the skills established in the regulations relating to the degree in nursing to obtain the degree that allows them to practice the profession. Competencies are demonstrated prior to professional service as a Registered Nurse but endure and develop throughout the professional career [37]. University education should prepare future professionals to acquire skills that allow them to join clinical care with safety and quality [33,38]. In addition, students must understand that their own spirituality will influence the care and relationship with patients [39]. Coping with health emergencies such as the COVID-19 pandemic has highlighted this reality and its impact on recently graduated professionals when they have joined health centres for clinical care [40,41,42].

In order for students to acquire these competences, nursing has conceptual and professional models that guide it in the acquisition of a disciplinary vision of care and its application in clinical practice. Among the conceptual models of nursing appears the philosophy of Jean Watson. This model of human care has a humanistic and altruistic character, where the nurse must connect with her inner spiritual condition through a genuine presence at the time of care. The nurse develops personally and professionally through self-knowledge, spiritual practice and the recognition of their own feelings. This exercise involves life experiences, imagining the feelings of others and, in addition, the study of one’s own values, beliefs, as well as the relationship with oneself, with others and with the world. Thus, the conceptual vision will have an impact on professional practice [4,43]. During clinical care, the nursing care reflects the competencies in spiritual and emotional care through the nursing care process, which has diagnosis or clinical judgment as its central axis. Each of the phases of this nursing process are recorded in the medical record using standardized languages such as NANDA-I, NOC and NIC [44,45]. Thus, the NANDA-I diagnoses of this dimension of care are shown in Table 1. Furthermore, students must acquire communication skills, ability in interpersonal relationships and intrapersonal development to ensure optimal integration into multidisciplinary teams and encourage reflection on their own spiritual and emotional influences. The purpose is to raise awareness about the importance of these areas of life and their impact on self-care and professional care.

In this regard, currently no framework of competencies or learning outcomes based on the nursing care process has been found, nor does it include aspects of self-knowledge and reflection on one’s own spirituality and emotions. Similar studies on spiritual and emotional care, approached from the literature branch of learning and teaching competencies, include reviews of the literature and designs to explore different perspectives of the people involved. Giske’s review of the literature included ten articles based on interviews, surveys or clinical cases, but none provided a catalogue of competencies or learning outcomes to assess learning progress [27]. On the other hand, the Grounded Theory design appears in two publications: a study from the perspective of 19 teachers, on understanding and teaching to assess, treat and evaluate spiritual care; and a study on the perspective of 42 students about their perception, concerns and, also, how to value and provide spiritual care [46,47]. This last manuscript states that “*Nurses need a wide range of competences to fulfil the nursing focus on holistic patient care*”, however, none of the studies provide a catalogue on this type of care [47].

For this purpose, this research uses as a basis the competency units that define the areas of functions that students will perform at the end of their studies [48]. Communication, reflection on oneself and relationships with other people were added to this reference. This framework provides a structure of six units: the first four refer to the phases of the nursing care process and the last two are cross-sectional on communication and intrapersonal development.

This type of curricular design based on the nursing care model allows a continuous increase in recognized competencies, while visualizing the professional role and, consequently, its health and well-being benefits for the population [37].

### Main Aim

The aim of this study is to define a catalogue of learning outcomes for competencies in spiritual and emotional care for undergraduate nursing students; a catalogue designed under an approach based on competency units that integrate the nursing care process, communication, interpersonal relationships and reflection on oneself.

## 2. Materials and Methods

### 2.1. Design

The study was a design and validation of a catalogue of learning outcomes related to spiritual and emotional care competencies.

The object of the study was the learning outcomes that allowed for the assessment of the spiritual and emotional care competencies of undergraduate nursing students. Specifically, the field of research was limited to the Degree in Nursing in Spain. In this regard, this research sought to design a catalogue from the perspective of health service academics and employers, both with experience in research and teaching.

The procedure applied a mixed methodology where a panel of experts performed the validation through the Delphi technique.

The mixed methodology combined a bibliographic search, analysis of legislation and consensus by a group of experts with a modified Delphi technique. The experts completed a survey with a quantitative and qualitative assessment. This mixed methodology allowed for a combination of the benefits of the different techniques and collecting the knowledge contained in the bibliography and teaching experts.

The study complied with the Good Reporting of a Mixed Methods Study (GRAMMS) checklist [49].

### 2.2. Procedure

The study had two different phases that included an initial proposal for a catalogue of learning outcomes and, subsequently, a validation phase focused on the relevance, priority and writing of the learning outcomes.

#### 2.2.1. Phase I—Proposal Design

The first phase aimed to design a catalogue of learning outcomes related to competencies in spiritual and emotional care for undergraduate students.

Three researchers elaborated the initial proposal: two men and one woman, with a mean age of 50.67 years. All of them had undergraduate and postgraduate teaching experience of more than five years, experience in the design of nursing care topics, research and clinical experience. During the study, one researcher held a managerial position in the regional health service and was a professor of the Degree in Nursing, another was a professor of the Degree in Nursing and evaluator of the quality of health degrees and, finally, the third researcher directed the clinical care and mentoring by nursing specialists. All of them were linked to university teaching.

The structure that organised the proposal was the six competency units: UC1 Assessment and diagnosis; UC2 Care planning; UC3 Nursing interventions; UC4 Evaluation and quality; CU5 Communication and interpersonal relationships; CU6 Knowledge and intrapersonal development of the student.

**Figure 1:**
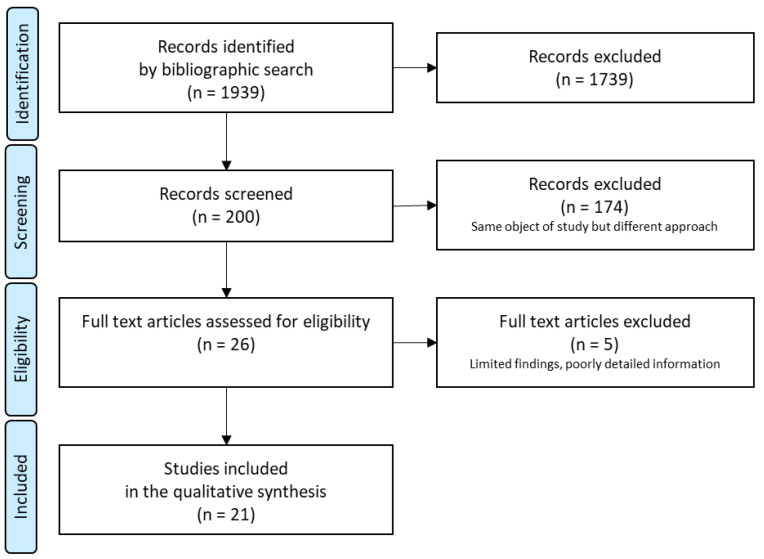
Flow diagram of analysis process.

The preliminary selection of the learning outcomes used a bibliographic review in MEDLINE, in Spanish and English languages and for the period 2014–2019. The terms were Schools, Nursing, Students, Nurse–Patient Relations, Professional–Patient Relations, Professional–Family Relations, Holistic Nursing, privacy, spirituality, Compassion Fatigue. The review followed the PRISMA statement of Preferred Reporting Items for Systematic Reviews and Meta-Analyses shown in Figure 1 [50].

In addition, the analysis included Spanish and European regulations for nursing training, such as Order CIN 2134/2008 and Directive 2013/55/EU of the European Parliament and of the Council [38,51].

The experts organised the results of the analysis of bibliographic sources in the competency units to align it to nursing practice. In this sense, the competency units related to the nursing care process considered other aspects.

The assessment used the Marjory Gordon patterns, selecting items related to spiritual assessment, religion, suffering and emotion [52]. The diagnostic phase integrated an analysis of the NANDA-I taxonomy in the domains of coping and stress tolerance, principles of life and self-perception to identify diagnoses related to spiritual and emotional care [44]. The diagnoses are summarized in Table 1. Similarly, the planning and intervention competency units used the NANDA-NOC-NIC links to guide the outcome criteria and interventions [45]. For this phase, the level of expertise of the undergraduate student was considered, so some proposals such as animal therapy, art therapy or music therapy interventions were excluded. The evaluation and quality were aimed at measuring the monitoring of the care process itself.

On the other hand, the competence unit of “Communication” addressed the information and confidentiality contained in the legislation and “Knowledge and intrapersonal development” addressed elements of self-knowledge and preventive measures against burnout or compassion fatigue.

The result was a catalogue of learning outcomes to assess competence in spiritual and emotional care organized under a structure of units oriented to practice and professional development.

#### 2.2.2. Phase II—Validation

The second phase aimed to validate the previously proposed catalogue of learning outcomes. The methodology used the modified Delphi technique in an online version with a panel of experts.

The participants of the expert panel were selected through purposive sampling. The selection applied the following criteria for a person to be considered an expert: more than 15 years of work experience in various fields related to the nursing profession, experience of undergraduate university training and currently working as a nurse in one of the following profiles recognized (clinical, management, teaching or research).

The modified Delphi technique was based on a survey sent by email and with two successive rounds for consensus. This technique maintained the anonymity of the participants and allowed interaction through comments and comparison with the group by providing feedback with measures of centrality of the set [53,54].

The principal investigator informed the participants about the aim, method and estimated time of the study. Once they agreed to participate, they were asked for their consent and an email for research correspondence.

Each participant individually received an email with the necessary information and the form with the survey to evaluate the learning results. The maximum response time was set at one month. Participants could only submit the entire survey once. In the event of an error in receiving the file, the valid survey was the last one received.

The survey showed the learning outcomes organised in the six competency units together with the columns to evaluate three criteria:

- Relevance: The expert considered the learning outcome appropriate and specific for its inclusion in the Nursing Degree curriculum;
- Priority: The expert considered the importance of demonstrating the learning outcome during the training process that enables one to practice the nursing profession;
- Clarity: The wording of the learning outcome was correct, clear, objective and consistent with the rest of the proposed outcomes.

The experts rated the first two aspects (relevance and priority) through a four-point Likert-type scale, where 1 was the minimum rating and 4 the most positive rating. This was a scale that did not allow neutral scores. For the clarity criterion, each expert was asked to indicate if they considered it appropriate to modify, join or eliminate any proposal, specifying in a cell the changes that they considered in free text. Finally, each expert had the opportunity to suggest new learning outcomes, either because they joined some of the original proposals or because they considered there were new aspects not covered.

To guarantee anonymity and data protection, all of the surveys were received by the same researcher (external to the design and analysis process) who verified that the files to be analysed did not contain data that would allow the participant to be identified. Once the files were downloaded and without identifying data, the surveys were sent to another researcher who analysed the responses and applied the analysis.

This researcher knew the aim of the research, the methodology and the purpose of applying the results. The expert was a nurse with a Ph.D. academic level, with more than ten years of clinical experience, teaching experience in Nursing Degrees and experience in the evaluation of Health Sciences degrees.

The analysis was quantitative for the relevance and priority criteria, and qualitative for the clarity and modification proposals.

The quantitative analysis applied the mean of the scores given by the participants for each learning outcome. Items with a score equal to or greater than 3.00 in relevance and priority were approved. Items with a score of less than 3.00 in relevance or priority were returned for evaluation in a second round, pending final consideration for validation or rejection.

At the same time, the qualitative analysis incorporated the contributions of the participants to modify the wording, join, eliminate or add items. These contributions were synthesised and shown in the second-round survey. Proposals for modifying the learning outcomes that obtained a score of 3.00 or more points in the criteria of relevance and priority were also included, that is, in those items that were approved in the first round but received improvements in the clarity of the wording.

The results of the analysis were sent to the group of three experts from the design phase, who sent the survey in the second round.

This second Delphi round included the catalogue of learning outcomes with the centrality scores of each item, the qualitative contributions and the individual assessment that each expert sent. Each expert analysed in the second round the point of view of the group together with the score awarded individually in the first round. In this phase, the type of responses was exclusively positive (accepted the change or proposal) or negative (rejected the proposal or modification).

The answers were treated as in the first round. First, the external researcher received the surveys and ensured anonymity and, later, the researcher for the analysis of results joined the responses to return the final consensus. 

The analysis of this second round included the final approval of those competencies that obtained a positive assessment from at least 80% of the experts, that is, from the entire group or from all but one.

The results were returned to the group of three experts who designed the initial catalogue and also to all of the study participants.

### 2.3. Ethics

The research did not use a sample of students and did not use a specific educational institution or health centre, so the ethical approval of the study by any centre of the participating experts was not necessary. However, this study was part of a doctoral dissertation at the Francisco de Vitoria University. The design of the study was presented to the academic managers and was positively valued for its realisation. The study did not imply any ethical consideration of special relevance. 

However, the methodology incorporated a panel of experts with different profiles and institutions. The principal investigator informed all these experts about the aim and methodology of the study, the conditions of anonymity and the purpose of disseminating the results. No expert presented a conflict of interest with the research, nor received any benefit for their participation.

All participants gave their informed consent for participation in the research and the use of data collected in the surveys.

## 3. Results

### 3.1. Phase I—Proposal Design

The initial proposal reached a total of 75 learning outcomes, distributed among the competency units as follows: 10 for Assessment and diagnosis, 5 for Planning, 27 for Intervention, 5 for Evaluation and quality, 11 for Communication and interpersonal relationships and finally, 17 for Knowledge and intrapersonal development of the student. 

The first unit of competence on Assessment and diagnosis integrates the basic aspects of knowledge, identification and prioritisation of nursing in relation to diagnoses.

In the unit of competence related to Planning, the focus is on the identification and planning of the clinical outcomes of care and the selection of interventions that allow the integration of spiritual and emotional needs in the plan of care [45]. These learning outcomes are of a medium–high level of difficulty [36].

The third unit of competence addresses the acquisition of knowledge, skills and attitudes that allow for the application of nursing interventions more related to spiritual and emotional care, such as counselling, relationships and facilitating self-forgiveness, among others that are found in the NIC taxonomy [45,55]. 

The learning outcomes related to Intervention are the most numerous, showing a wide range of action skills that the student must demonstrate in the practice of care. Under this framework, the fourth competency unit collects those results of student learning about knowledge of the tools for intervention and the skills for their use.

The fifth unit, Communication and interpersonal relationship, focuses on the creation of a communication environment that allows the expression of people’s feelings and emotions and, in addition, ensures that their values and beliefs are respected, showing hospitality and creating a climate of intimacy.

The last competency unit presents a higher level of complexity, because it requires a personal maturity aimed at self-knowledge and self-care of the student.

The complete catalogue of learning outcomes proposed in the first phase appears in Table S1, in the first column of the table.

### 3.2. Phase II—Validation

All of the participants of the expert group answered the surveys in both phases. The expert panel included seven nurses with the demographic characteristics shown in Table 2.

The evaluation of the learning outcomes appears in Table S1, the initial catalogue and the relevance and priority score, and in Table S2, the comparison between the initial and final catalogue of learning outcomes. The experts evaluated the 75 proposed learning outcomes. Relevance obtained an average score of 3.54, with a minimum of 2.83 and a maximum of 4.00. Only two items obtained a mean score less than 3.00. The priority obtained an average of 3.37, with a minimum of 2.67 and a maximum of 4.00. Only nine items obtained a mean score lower than 3.00.

However, during the first-round, multiple contributions appeared. The researcher who analysed the surveys joined them and integrated them into the survey for validation in the second-round.

After the second round, the experts validated seven learning outcomes of the new proposals: four for Assessment and diagnosis, one for Planning and two for Communication and interpersonal relationships. On the contrary, 17 items were eliminated because they were not considered appropriate for the purpose and scope of application or because they were unified in other learning outcomes. The change in the learning outcome focused on the right to information that the experts transferred to the Planning competence unit stands out, considering that the professionals take this right into account from the beginning of the care process.

In addition, the experts modified the wording of 36 learning outcomes: 6 for Assessment and diagnosis, 2 for planning, 14 for Intervention, 4 for Evaluation and quality, 4 for Communication and interpersonal relationships and, finally, 6 for Knowledge and intrapersonal development of the student. Most of the changes were related to the standardisation of the wording. The experts approved using the term “person” instead of varying between “patient” or “individual” and eliminated the concept of “intimate care” because it is not developed in the literature. They preferred “spiritual and emotional care” or each area separately when they considered it so.

The experts eliminated from the final catalogue some of the learning outcomes that obtained a score greater than 3.00 in the first round because they considered that their meaning was already included in other learning outcomes after modifying the clarity of their wording between the first and second rounds.

Ultimately, the experts approved 65 learning outcomes.

#### 3.2.1. Assessment and Diagnosis

The initial proposal included 10 learning outcomes. The experts modified six, did not eliminate any, and included four new items.

Finally, the group validated 14 learning outcomes.

- Identify the dimensions that spirituality encompasses, differentiating between spirituality and religion;
- Perform a conceptualisation of the spiritual and emotional sphere of the person;
- Carry out an assessment of the needs related to the spiritual and emotional area;
- Show respect and closeness during the assessment of the person, creating an environment that is favourable to communication;
- Recognise that the illness may affect the person’s values and beliefs;
- Detect the presence of suffering in the person;
- Assess the person’s spiritual well-being;
- Perform a priority analysis on the information collected;
- Identify nursing diagnoses related to the spiritual area;
- Identify nursing diagnoses related to the emotional area;
- Know the defining characteristics and related factors/ risk factors of nursing diagnoses related to the spiritual and emotional area;
- Carry out a differential diagnosis between the different care problems in the spiritual area;
- Carry out a differential diagnosis between the different care problems in the emotional area;
- Make the record of the assessment and diagnosis of the spiritual and emotional area.

#### 3.2.2. Planning

The initial proposal included five learning outcomes. The experts modified two, eliminated one and included another new element.

Finally, the group validated five learning outcomes.

- Select the outcomes criteria for each person taking into account their overall situation and their values and beliefs;
- Select the interventions related to the spiritual and emotional area, establishing an order of priority;
- Make a record of the planned care plan and the expected evolution of the person;
- Carry out a care plan focused on coping with threats that may increase suffering;

Identify the person’s need for information, respecting the right to decide about it.

#### 3.2.3. Intervention

The initial proposal included 27 learning outcomes. The experts modified 13, eliminated 10 and did not propose new learning outcomes. This competence unit presents a greater number of initial proposals and later modifications. In general, the experts considered it opportune to unify items that shared a focus of interest in the evaluation. On the other hand, the experts did not approve the learning outcomes of grief, considering it a vital process where spirituality and emotionality are involved, but which deserves separate analysis.

Finally, the group validated 17 learning outcomes.

- Dedicate time to the relationship with the person being cared for, maintaining continuity in the relationship;
- Respect the needs and demands of privacy of the person, respecting moments of silence and solitude and moments of meeting with loved ones;
- Facilitate the expression of feelings of guilt and forgiveness, identifying the painful feelings of guilt and directing the person in self-forgiveness;
- Facilitate the spiritual growth of the person and their family, helping them to explore beliefs in relation to healing;
- Facilitate religious practice, encouraging conversation about their interests, use and participation in rituals or practices that do not harm health;
- Help the person in the acceptance and search for meaning in life;
- Help the person recognise and express feelings such as anxiety, anger, or sadness;
- Listen to the expression of feelings about the loss;
- Help the person to control anger by identifying its causes, developing appropriate methods of expression and training in techniques that provide calm;
- Help the person to enhance self-esteem, encouraging positive statements about oneself and facilitating an environment and activities that increase self-esteem;
- Help the person to train assertiveness, monitoring levels of anxiety and discomfort related to behaviour change;
- Help the person clarify the values and expectations that may be involved in making life decisions;
- Help the person and their family to identify the areas of hope in life, reviewing the goals related to the object of hope and including them in the care plan;
- Apply the counselling technique, helping the person to identify the problem or related factor, prioritising possible alternatives to the problem, considering their strengths and weaknesses;
- Apply relaxation techniques;
- Give emotional support to the person;

Maintain the confidentiality of the person’s health information.

#### 3.2.4. Evaluation and Quality

The initial proposal included five learning outcomes. The experts modified four, eliminated one and did not propose new ones. 

The learning outcomes “*Assess the evolution of the patient’s healing process during the care process*” was eliminated for the same reason that was eliminated in the Planning competency unit, that is, the complexity and implications of the concept “healing”.

Finally, the group validated four learning outcomes.

- Monitor the person’s spiritual and emotional situation, through the selected indicators;
- Monitor the person’s level of suffering through the selected indicators;
- Evaluate the impact of care on the level of suffering of the person;

Implement improvement actions based on the results in care of the spiritual and emotional area, adapting the interventions of the care plan when necessary.

#### 3.2.5. Communication and Interpersonal Relationship

The initial proposal included 11 learning outcomes. The experts modified four, eliminated five and proposed two new items. Finally, the group validated eight learning outcomes.

- Show hospitality in welcoming the person, showing interest in their values and expectations;
- Create a climate of intimacy that allows communication on aspects of the spiritual and emotional area of the person;
- Identify the situations in which the person requires spaces of silence and respect them;
- Transmit truthfulness and use clear language without hesitation, responding to the person’s doubts;
- Do not make judgments and respect the ontological dignity of the person when the values and beliefs are different from their own;
- Plan care considering the moments of intimacy of the person;
- Listen to the expression of feelings about the loss;
- Carry out active listening avoiding barriers and using silence/listening to encourage expressing feelings, thoughts and concerns.

#### 3.2.6. Knowledge and Intrapersonal Development of the Student

The initial proposal included 17 learning outcomes. The experts modified six and did not eliminate or propose new items.

Finally, the group validated the 17 learning outcomes.

- Reflect on one’s own vocation, vital values and attitudes, identifying positive and negative attitudes towards caring for the spiritual and emotional area;
- Reflect on one’s own values and beliefs and identify how they influence caring for others;
- Recognise one’s own limits and virtues in spiritual care;
- Recognise the importance of spirituality in your life;
- Recognise the signs of spiritual and emotional exhaustion;
- Show personal knowledge by analysing one’s own strengths and weaknesses on a spiritual and emotional level;
- Show self-awareness and emotional control, maintaining self-control in situations of personal suffering;
- Show a proactive attitude of improvement on a personal level;
- Find spaces to reflect and connect with yourself: meditation, directed imagination, relaxation;
- Find solutions to the negative influence of one’s own values and beliefs in care;
- Identify situations that cause stress;
- Identify the signs and symptoms of “compassion fatigue” or “cost of caring”;
- Relate sensations and experiences in stressful situations to the team;
- Analyse how affects the situations of the people cared to one’s inner life and relationships;
- Respect values and beliefs other than your own;
- Ask for help in situations that you cannot control or resolve;
- Learn to treat spiritual and emotional care problems identified in the person as a team.

## 4. Discussion

Nursing university training encompasses holistic care for all areas of the person, including the spiritual area and its expression in the emotional area [56]. Nursing programmes must include competencies for the spiritual and emotional care of the student because, depending on their own vision of the world, the future professional will be able to provide culturally congruent care to any person or population [21,29].

These aspects are fundamental for university education and the code of professional ethics [7,51].

Under this framework, the present study has focused on a set of competency units oriented to spiritual and emotional care. The learning outcomes are aimed at providing patient care, as well as the student’s reflection on their abilities and limitations.

Similar researchers have addressed this area and have shown the importance of defining a framework of competencies to be achieved [20,35,48,57,58,59,60,61]. Some manuscripts highlighted the main areas for learning spiritual care and the dimensions of spiritual care practice [27,28]. Other studies explore the effectiveness of an intervention programme to improve students’ ability to assess spirituality [56]. On the other hand, published articles showed the use of tools to assess the spirituality of patients, such as the Faith, Importance and Influence, Community, and Address (FICA) Spiritual History Tool, and changes in students’ perception and provision of spiritual care, such as the Spirituality and Spiritual Care Rating Scale (SSCRS) [29]. Furthermore, the research found pointed out the importance of reflection and self-knowledge, of learning from the analysis of experiences and, in addition, the requirement to develop care plans that include the assessment of the spiritual area [29,46].

Even today, there is no extended and implemented common frame of reference. However, spiritual and emotional care competencies must be adapted to the society, culture and regulations of each region. In this sense, the explicit inclusion of terms and competencies related to spirituality and emotion are related to better results in awareness, knowledge, skills and attitudes in situations of spiritual care [22,57].

This research focuses on undergraduate students and applies a structure of competency units as a framework, which includes a nursing care process model. These characteristics differentiate this research from similar proposals that investigate spiritual care, the care process and communication, but focused exclusively on the perception of professionals and without applying a reference nursing model [21,23,25].

Likewise, the purpose of this study is the creation of a catalogue of specific and concrete learning outcomes that can be implemented in any university degree programme in nursing, fully or partially, for the configuration of competencies in spiritual and emotional care.

The numerous contributions of the participants show the complexity of formally specifying the learning outcomes of this dimension of care. Among the contributions provided, the unification of learning outcomes oriented to the same focus stands out, which reduces the initial number of proposals that represented a similar meaning. However, in the first round, contributions of new learning outcomes were also received. It highlights the importance of active listening in communication as an implicit basis in the entire competence unit that the experts make explicit in learning outcomes.

Furthermore, between the first and second rounds, the study homogenised the subject of the proposed sentences under the term “person”. The experts proposed to eliminate the variety of terms such as “individual” or “patient” and use the term “person” because it is more appropriate to reflect a comprehensive vision of care, which includes the situation of hospitalisation as well as care in the home environment or in the community. This change is in accordance with the *Knowledge Model about Person Care* [62]. 

Once the study integrated the changes proposed by the experts in the first round, the results were sent back to the panel. The second round obtained the consensus of the group and there were no further suggestions for elimination or new proposals.

No similar published study includes this category. The study establishes the analysis and recognition by the student of their values, beliefs and fears as an essential competence to be able to establish a helping relationship with any person cared for. The student must know themselves well, recognise situations such as compassion fatigue and be prepared to face them by learning to find spaces to stop and connect with themselves through meditation, directed imagination or relaxation.

The nurse’s attitude toward the patient’s spirituality is important to the provision of spiritual care. The way nurses describe their own spirituality is a predictor of the quality of spiritual care they will provide [60].

In parallel, coping with self-destructive behaviours and deficits in the self-care of the professionals contribute to the elevation of stress levels, depression and burnout in students. Possessing compassion and learning compassion are identified as a means of protection against stress [63].

With regard to students, Meyer discusses their spirituality and the emphasis on this area of care during their education as an important predictor of spiritual care [48]. Programmes such as that of the University of Isafahan focus on the development of competencies that help the student cope with situations of stress and anxiety to achieve emotional wellbeing [64]. The training includes relaxation techniques, association between thoughts and emotions or even logical thought processes. The findings of this training were positive.

Other training programmes for healthcare students that have demonstrated their effectiveness employ mindfulness with the intention of reducing factors such as compassion, fatigue, stress or the anxiety that leads to burnout [65,66,67].

The study concurs with other works that indicate the importance of including topics related to the emotional and spiritual care of the patient, such as personal self-care, into the nursing curriculum. Both students and professionals find problems with addressing the care of these parts of the patient and of the problems that are derived from it. Amongst the reasons for these difficulties is the lack of training during the university stage, which reinforces the need to define learning outcomes to train future nurses in these care competencies [65,66,67].

### 4.1. Limitations and Future Lines

The findings of this study must be closely analysed before being extrapolated to other environments. In order for this to occur, it is essential for the limits of this research to be considered as well.

The selected learning outcomes have been designed and validated for the field of undergraduate nursing education in Spain, which has its own specific regulatory framework [51]. This framework can serve as a base for other regions but must be analysed and adapted to the specific regulations of each country and institution.

On the other hand, this methodology has employed a limited number of experts with profiles that are academic (but also one clinical one) in a similar way to previous research on competency design [68]. This profile is sought after because the experts on the panel are familiar with the regulations, have teaching experience and know the correct way to write and assess the learning outcomes. This panel could be amplified in other studies to include clinical experts who participate in practical teaching and by a representation of students to include their considerations.

In the future, the designed learning outcomes should be evaluated for application in real theoretical and practical training environments. Some questions that could be addressed are whether the learning results meet the purpose of evaluating professional competence in spiritual and emotional care, whether the implementation of this catalogue influences generating greater attention to these areas of the programmes or, among other ideas, if this inclusion modifies the importance that students give to spiritual and emotional care.

### 4.2. Relevance to Clinical Practice

The potential benefits include teachers and students. University professors and clinical practice tutors know what students should work on and can help them in personal reflection and in the reflection of practical cases. Consequently, students more clearly identify the knowledge, skills or attitudes that they are expected to demonstrate. The student can know what is required in their learning, can reflect on the quality of care that is being provided and, additionally, can find the opportunity to reflect on their own limitations in spiritual and emotional care. This benefit in university education may be reflected in clinical practice because it seeks to establish a process of continuous reflection and improvement in the area of spiritual and emotional care. In this sense, Jean Watson’s philosophical model already pointed out the importance of genuine presence in care and the transcendence of spirituality and emotion in the relationship with the patient being cared for, both for the patient and for the professional. Thus, this study translates this nursing philosophy and makes it operational through a catalogue of learning outcomes that make explicit a learning–teaching framework. Care goes beyond the art of the act of care, of technical expertise, because it is completely influenced by a humanistic vision that gives meaning to the relationship with the person. A significant relationship that seeks to improve patient satisfaction and, in addition, the satisfaction of the professional who finds a transpersonal condition in the world and updates his ontological competence through caring for the other [4,43].

## 5. Conclusions

The study achieved the objective of defining a catalogue of learning outcomes that help in the evaluation of undergraduate nurses to provide them with knowledge, skills and attitudes focused on providing people with comprehensive care that includes the spiritual and emotional dimensions.

The implementation of this framework must be linked to the design of an academic plan that includes theoretical and practical content throughout different academic years, from low complexity to high complexity.

## Figures and Tables

**Table 1:** NANDA-I Diagnoses for spiritual and emotional care.

Code	Diagnostic Label
00148	Fear
00124	Hopelessness
00066	Spiritual distress
00169	Impaired religiosity
00170	Risk for impaired religiosity
00054	Risk for loneliness
00147	Death anxiety
00120	Situational low self-esteem
00119	Chronic low self-esteem
00174	Risk for compromised human dignity
00214	Impaired comfort
00136	Grieving
00135	Complicated grieving
00172	Risk for complicated grieving
00121	Readiness for enhanced hope
00210	Impaired resilience
00175	Moral distress
00137	Chronic sorrow

**Table 2:** Characteristics of expert panel.

Age (Years)	Average (Range)	51 (40–62)
Sex (*n*)	Female	6
	Male	1
Clinical Experience (years)		>15
Professional Profile (*n*)	Clinical	1
	Teaching	4
	Management	4
	Research	5
Academic Background (*n*)	PhD	5
	Master	2
Professional Experience (*n*)	Clinical	7
	Teaching	5
	Management	4
	Research	7
Teaching Experience (*n*)	Undergraduate	5
	Post-graduate	5
	Other teaching experience	2

## Data Availability

The data that support the findings of this study are available on request from the corresponding author. The data are not publicly available due to privacy or ethical restrictions.

## Supplementary Materials

The following supporting information can be downloaded at: https://www.mdpi.com/article/10.3390/healthcare10102062/s1, Table S1: Evaluation of proposed learning outcomes: Round 1. Table S2: Learning outcomes initially proposed and validated at the end of Round 2.
