# Supplementary material for: Competence in Spiritual and Emotional Care: Learning Outcomes for the Evaluation of Nursing Students
Source: Healthcare (Basel). 2022 Oct 17;10(10):2062. doi: 10.3390/healthcare10102062 (PMC9601644; doi:10.3390/healthcare10102062)
Supplement: Supplementary file 1 [file healthcare-10-02062-s001.zip › Table S1 REV.pdf]

healthcare MDPI

Table S1. Evaluation of proposed learning outcomes: Round 1.

	Relevance				Priority			
**1. Assessment and diagnosis**	**Mean**	Median	Mode	Range	**Mean**	Median	Mode	Range
Identify the dimensions that spirituality encompasses, differentiating between spirituality and religion.	**3.43**	4.00	4.00	2-4	**2.86**	3.00	3.00	2-4
Carry out a correct assessment of the patterns/needs related to the spiritual and emotional area.	**4.00**	4.00	4.00	4-4	**3.43**	3.00	3.00	3-4
Show respect and closeness during the assessment of the person, creating an environment that is favourable to communication.	**3.57**	4.00	4.00	3-4	**3.86**	4.00	4.00	3-4
Recognise that the illness may affect the patient's values and beliefs.	**3.57**	4.00	4.00	3-4	**3.43**	3.00	3.00	3-4
Detect the presence of suffering in the person.	**3.86**	4.00	4.00	3-4	**3.71**	4.00	4.00	3-4
Perform a priority analysis on the information collected.	**3.57**	4.00	4.00	3-4	**3.43**	4.00	4.00	2-4
Know the nursing diagnoses that affect the intimacy of the person being cared for.	**3.57**	4.00	4.00	3-4	**3.71**	4.00	4.00	3-4
Identify diagnoses related to the spiritual and emotional area of the patient.	**3.57**	4.00	4.00	3-4	**3.71**	4.00	4.00	3-4
Know the defining characteristics and related factors/ risk factors of nursing diagnoses related to patient intimacy.	**3.57**	4.00	4.00	3-4	**3.71**	4.00	4.00	3-4
Make a correct record of everything identified.	**3.83**	4.00	4.00	3-4	**3.67**	4.00	4.00	3-4
**2. Planification**								
Select the outcomes criteria for each person taking into account their overall situation and their values and beliefs.	**3.71**	4.00	4.00	3-4	**3.71**	4.00	4.00	3-4
Select the interventions related to the spiritual and emotional area, establishing an order of priority.	**3.71**	4.00	4.00	3-4	**4.00**	4.00	4.00	4-4
Make a record of the executed care plan and the evolution of the patient.	**3.71**	4.00	4.00	3-4	**3.57**	4.00	4.00	3-4
Carry out a care plan focused on coping with threats that may increase suffering, if they exist.	**3.14**	3.00	3.00	2-4	**3.14**	3.00	3.00	2-4
Include in the care plan interventions that contribute to the healing process.	**3.00**	3.00	3.00	2-4	**2.71**	3.00	3.00	2-4

**3. Intervention**								
Dedicate time to the relationship with the person being cared for, maintaining continuity in the relationship.	**3.71**	4.00	4.00	3-4	**3.71**	4.00	4.00	3-4
Respect the needs and demands of privacy of the person, respecting moments of silence and solitude and moments of meeting with loved ones.	**3.57**	4.00	4.00	3-4	**3.57**	4.00	4.00	3-4
Facilitate the expression of feelings of guilt and forgiveness by identifying painful feelings of guilt and leading the patient through the steps of self-forgiveness when self-blame is valid.	**3.00**	3.00	3.00	2-4	**2.86**	3.00	3.00	2-4
Facilitate the spiritual growth of the person being cared for and their family by helping them to explore beliefs in relation to the healing of the body, mind and spirit and provide an environment that favours a meditative / contemplative attitude for self-reflection	**3.14**	3.00	4.00	2-4	**2.86**	2.00	2.00	2-4
Facilitate religious practice by encouraging the use of and participation in any religious ritual or practice that is not detrimental to health and discussion of religious interests.	**3.50**	3.50	4.00	3-4	**3.00**	3.00	4.00	2-4
Help the patient in the acceptance and search for meaning in her life.	**3.40**	4.00	4.00	2-4	**3.00**	3.00	3.00	2-4
Help the patient recognise and express feelings such as anxiety, anger, or sadness.	**3.60**	4.00	4.00	3-4	**3.60**	4.00	4.00	3-4
Listen to expressions of grief and expression of feelings about the loss.	**3.60**	4.00	4.00	3-4	**3.60**	4.00	4.00	3-4
Help the patient to control anger through the identification of the causes, the development of adequate methods of expression of the same and the instruction in the measures that provide calm.	**3.17**	3.00	3.00	2-4	**2.83**	3.00	3.00	2-4
Help the person cared for to enhance their self-esteem by providing an environment and activities that increase self-esteem and making positive statements about them.	**3.43**	3.00	3.00	3-4	**3.14**	3.00	3.00	2-4
Help the patient to train her assertiveness through strategies for the practice of assertive behaviour, monitoring the levels of anxiety and discomfort related to behaviour change.	**3.50**	3.50	3.00	3-4	**3.00**	3.00	3.00	2-4
Help the person clarify the values and expectations that may be involved in making life decisions.	**3.43**	3.00	3.00	3-4	**3.29**	3.00	3.00	2-4
Help the cared person/family to identify the areas of hope in life and to design and review the goals related to the object of hope, including	**3.14**	3.00	3.00	2-4	**3.00**	3.00	3.00	2-4

them in the care plan and promoting therapeutic relationships with loved ones.								
Correctly apply the counselling technique, helping the patient to identify the problem or related factors, prioritising possible alternatives to the problem, considering their strengths and weaknesses.	**3.50**	3.50	4.00	3-4	**3.33**	3.00	3.00	3-4
Apply relaxation techniques, assessing, planning, and evaluating the development and the result of it.	**3.50**	3.50	3.00	3-4	**3.00**	3.00	3.00	2-4
Implement actions, within the care plan, to provide emotional support to the person being cared for.	**3.33**	3.00	3.00	3-4	**3.33**	3.00	3.00	3-4
Maintain the confidentiality of patient health information.	**4.00**	4.00	4.00	4-4	**3.71**	4.00	4.00	2-4
Teach the person relaxation techniques.	**3.50**	3.50	3.00	3-4	**2.75**	3.00	3.00	2-4
Carry out active listening avoiding barriers and using silence/listening to encourage expressing feelings, thoughts and concerns.	**3.86**	4.00	4.00	3-4	**3.43**	3.00	3.00	3-4
Support the patient in making decisions by informing the patient about the existence of alternative points of view and solutions clearly and with full support.	**3.50**	3.50	3.00	3-4	**3.17**	3.00	3.00	2-4
Provide the information requested by the patient respecting the patient's right to receive or not receive information.	**3.57**	4.00	4.00	1-4	**3.43**	4.00	4.00	1-4
Serve as a connection between the patient and family and with other health professionals.	**3.29**	3.00	3.00	2-4	**3.29**	3.00	3.00	2-4
Identify the situations in which the person being cared for may require spiritual support, implementing actions, within the care plan, to provide spiritual support to the person being cared for.	**3.83**	4.00	4.00	3-4	**3.50**	4.00	4.00	2-4
Provide an environment that promotes private conversations between the patient, family and health professionals.	**3.33**	3.50	4.00	2-4	**3.00**	3.00	3.00	2-4
Implement actions to alleviate suffering.	**3.67**	4.00	4.00	3-4	**3.33**	3.50	4.00	2-4
Identify the situation of the person's grieving process: denial, anger, negotiation, depression and acceptance.	**3.67**	4.00	4.00	3-4	**3.67**	4.00	4.00	3-4
Support progression through personal stages of grief based on the person's situation.	**2.83**	3.00	3.00	2-4	**2.67**	2.50	2.00	2-4
**4. Evaluation and quality**								
Monitor the patient's spiritual and emotional situation, through the selected indicators.	**3.67**	4.00	4.00	3-4	**3.50**	3.50	3.00	3-4

Monitor the level of suffering, through the selected indicators.	**3.50**	3.50	3.00	3-4	**3.33**	3.00	3.00	3-4
Evaluate the impact of care on their level of suffering.	**3.33**	3.00	3.00	3-4	**3.50**	3.50	3.00	3-4
Implement improvement actions based on the results, adapting the interventions of the care plan when necessary.	**3.67**	4.00	4.00	3-4	**3.67**	4.00	4.00	3-4
Assess the evolution of the patient's healing process during the care process.	**3.00**	3.00	3.00	2-4	**3.00**	3.00	3.00	2-4
**5. Communication and interpersonal relationship**								
Show hospitality in welcoming the person, showing interest in their values and expectations.	**4.00**	4.00	4.00	4-4	**4.00**	4.00	4.00	4-4
Create a climate of intimacy that allows communication on aspects of the spiritual and emotional area of the person.	**3.57**	4.00	4.00	3-4	**3.57**	4.00	4.00	3-4
Identify the situations in which the patient requires spaces of silence and respect them.	**3.57**	4.00	4.00	3-4	**3.29**	3.00	3.00	3-4
Convey truthfulness, use frank language without hesitation, responding to the patient's doubts.	**3.71**	4.00	4.00	3-4	**3.43**	4.00	4.00	2-4
Respect and not judge the ontological dignity of the patient, values and beliefs that may be different from their own.	**3.83**	4.00	4.00	3-4	**3.83**	4.00	4.00	3-4
Plan care considering the moments of intimacy of the patient.	**3.29**	4.00	4.00	3-4	**3.00**	3.00	4.00	2-4
Respect the confidentiality of the information.	**4.00**	4.00	4.00	4-4	**3.86**	4.00	4.00	3-4
Offer help to the person cared for to collaborate in the resolution of suffering.	**3.57**	4.00	4.00	3-4	**3.29**	3.00	4.00	2-4
Implement actions aimed at alleviating suffering.	**3.83**	4.00	4.00	3-4	**3.50**	3.50	4.00	3-4
Plan care respecting the values and beliefs of the person being cared for and their environment.	**3.86**	4.00	4.00	3-4	**3.86**	4.00	4.00	3-4
Respect the patient's right not to know.	**3.71**	4.00	4.00	3-4	**3.71**	4.00	4.00	3-4
**6. Knowledge and intrapersonal development of the student**								
Reflect on one's own vocation, vital values and attitudes, identifying positive and negative attitudes towards the care of the intimate.	**3.43**	3.00	3.00	3-4	**3.14**	3.00	3.00	2-4
Reflect on one's own values and beliefs and identify how they influence caring for others.	**3.71**	4.00	4.00	3-4	**3.86**	4.00	4.00	3-4
Recognise one's own limits and virtues in spiritual care.	**3.57**	4.00	4.00	3-4	**3.43**	3.00	3.00	3-4
Recognise the importance of spirituality in your life.	**3.57**	4.00	4.00	3-4	**3.00**	3.00	3.00	2-4

Recognise the signs of emotional, psychic and spiritual exhaustion.	**3.71**	4.00	4.00	3-4	**3.29**	3.00	3.00	2-4
Show personal knowledge by analysing one's own strengths and weaknesses on a spiritual and psychological level.	**3.60**	4.00	4.00	3-4	**3.20**	3.00	3.00	2-4
Show self-awareness and emotional control, maintaining self-control in situations of personal suffering.	**3.71**	4.00	4.00	3-4	**3.57**	4.00	4.00	3-4
Show a proactive attitude of improvement on a personal level.	**3.71**	4.00	4.00	3-4	**3.71**	4.00	4.00	3-4
Find spaces to stop and connect with yourself: meditation, guided imagery, relaxation.	**3.14**	3.00	4.00	2-4	**2.86**	3.00	2.00	2-4
Find solutions to the negative influence of one's own values and beliefs in care.	**3.83**	4.00	4.00	3-4	**3.67**	4.00	4.00	3-4
Identify situations that cause stress.	**3.50**	3.50	3.00	3-4	**3.50**	3.50	3.00	3-4
Identify the signs and symptoms of “compassion fatigue” or “cost of caring”.	**3.29**	3.00	3.00	2-4	**3.14**	3.00	3.00	2-4
Relate sensations and experiences in stressful situations to the team.	**2.86**	3.00	3.00	2-4	**2.71**	3.00	3.00	2-4
Analyse how the situations of patients affect their own inner life and relationships.	**3.29**	3.00	3.00	3-4	**3.00**	3.00	3.00	2-4
Respect values and beliefs other than your own.	**3.86**	4.00	4.00	3-4	**3.71**	4.00	4.00	3-4
Ask for help in situations that you cannot control or resolve.	**3.71**	4.00	4.00	3-4	**3.57**	4.00	4.00	3-4
Learn to treat in a team the intimacy problems identified in the patient.	**3.43**	3.00	3.00	3-4	**3.29**	3.00	3.00	3-4
