# Supplementary material for: Competence in Spiritual and Emotional Care: Learning Outcomes for the Evaluation of Nursing Students
Source: Healthcare (Basel). 2022 Oct 17;10(10):2062. doi: 10.3390/healthcare10102062 (PMC9601644; doi:10.3390/healthcare10102062)
Supplement: Supplementary file 1 [file healthcare-10-02062-s001.zip › Table S2 REV.pdf]

healthcare

MDPI

Table S2. Learning outcomes initially proposed and validated at the end of Round 2

Round 1. Proposed learning outcomes	Round 2. Validated learning outcomes	Changes
**1. Assessment and diagnosis**		
Identify the dimensions that spirituality encompasses, differentiating between spirituality and religion.	Identify the dimensions that spirituality encompasses, differentiating between spirituality and religion.	
	Perform a conceptualisation of the spiritual and emotional sphere of the person.	New
Carry out a correct assessment of the patterns/needs related to the spiritual and emotional area.	Carry out an assessment of the needs related to the spiritual and emotional area.	Modified
Show respect and closeness during the assessment of the person, creating an environment that is favourable to communication.	Show respect and closeness during the assessment of the person, creating an environment that is favourable to communication.	
Recognize that the illness may affect the patient's values and beliefs.	Recognise that the illness may affect the person's values and beliefs.	Modified
Detect the presence of suffering in the person.	Detect the presence of suffering in the person.	
	Assess the person's spiritual well-being.	New
Perform a priority analysis on the information collected.	Perform a priority analysis on the information collected.	
Know the nursing diagnoses that affect the intimacy of the person being cared for.	Identify nursing diagnoses related to the spiritual area.	Modified
Identify diagnoses related to the spiritual and emotional area of the patient.	Identify nursing diagnoses related to the emotional area.	Modified
Know the defining characteristics and related factors/ risk factors of nursing diagnoses related to patient intimacy.	Know the defining characteristics and related factors/ risk factors of nursing diagnoses related to the spiritual and emotional area.	Modified
	Carry out a differential diagnosis between the different care problems in the spiritual area.	New
	Carry out a differential diagnosis between the different care problems in the emotional area.	New
Make a correct record of everything identified.	Make the record of the assessment and diagnosis of the spiritual and emotional area.	Modified
**2. Planification**		
Select the outcomes criteria for each person taking into account their overall situation and their values and beliefs.	Select the outcomes criteria for each person taking into account their overall situation and their values and beliefs.	
Select the interventions related to the spiritual and emotional area, establishing an order of priority.	Select the interventions related to the spiritual and emotional area, establishing an order of priority.	

Make a record of the executed care plan and the evolution of the patient.	Make a record of the planned care plan and the expected evolution of the person.	Modified
Carry out a care plan focused on coping with threats that may increase suffering, if they exist.	Carry out a care plan focused on coping with threats that may increase suffering.	Modified
	Identify the person's need for information, respecting the right to decide about it.	New
Include in the care plan interventions that contribute to the healing process.		Removed
**3. Intervention**		
Dedicate time to the relationship with the person being cared for, maintaining continuity in the relationship.	Dedicate time to the relationship with the person being cared for, maintaining continuity in the relationship.	
Respect the needs and demands of privacy of the person, respecting moments of silence and solitude and moments of meeting with loved ones.	Respect the needs and demands of privacy of the person, respecting moments of silence and solitude and moments of meeting with loved ones.	
Facilitate the expression of feelings of guilt and forgiveness by identifying painful feelings of guilt and leading the patient through the steps of self-forgiveness when self-blame is valid.	Facilitate the expression of feelings of guilt and forgiveness, identifying the painful feelings of guilt and directing the person in self-forgiveness.	Modified
Facilitate the spiritual growth of the person being cared for and their family by helping them to explore beliefs in relation to the healing of the body, mind and spirit and provide an environment that favours a meditative / contemplative attitude for self-reflection	Facilitate the spiritual growth of the person and their family, helping them to explore beliefs in relation to healing.	Modified
Facilitate religious practice by encouraging the use of and participation in any religious ritual or practice that is not detrimental to health and discussion of religious interests.	Facilitate religious practice, encouraging conversation about their interests, use and participation in rituals or practices that do not harm health.	Modified
Help the patient in the acceptance and search for meaning in life.	Help the person in the acceptance and search for meaning in life.	Modified
Help the patient recognize and express feelings such as anxiety, anger, or sadness.	Help the person recognise and express feelings such as anxiety, anger, or sadness.	Modified
Listen to expressions of grief and expression of feelings about the loss.	Listen to the expression of feelings about the loss.	Modified
Help the patient to control anger by identifying its causes, developing appropriate methods of expression and the instruction in the measures that provide calm.	Help the person to control anger by identifying its causes, developing appropriate methods of expression and training in techniques that provide calm.	Modified
Help the person cared for to enhance self-esteem by providing an environment and activities that increase self-esteem and making positive statements about oneself.	Help the person to enhance self-esteem, encouraging positive statements about oneself and facilitating an environment and activities that increase self-esteem.	Modified

Help the patient to train assertiveness through strategies for the practice of assertive behaviour, monitoring the levels of anxiety and discomfort related to behaviour change.	Help the person to train assertiveness, monitoring levels of anxiety and discomfort related to behaviour change.	Modified
Help the person clarify the values and expectations that may be involved in making life decisions.	Help the person clarify the values and expectations that may be involved in making life decisions.	
Help the cared person/family to identify the areas of hope in life and to design and review the goals related to the object of hope, including them in the care plan and promoting therapeutic relationships with loved ones.	Help the person and their family to identify the areas of hope in life, reviewing the goals related to the object of hope and including them in the care plan.	Modified
Correctly apply the counselling technique, helping the patient to identify the problem or related factors, prioritizing possible alternatives to the problem, considering their strengths and weaknesses.	Apply the counselling technique, helping the person to identify the problem or related factor, prioritising possible alternatives to the problem, considering their strengths and weaknesses.	Modified
Apply relaxation techniques, assessing, planning, and evaluating the development and the result of it.	Apply relaxation techniques.	Modified
Implement actions, within the care plan, to provide emotional support to the person being cared for.	Give emotional support to the person.	Modified
Maintain the confidentiality of patient health information.	Maintain the confidentiality of the person's health information.	Modified
Teach the person relaxation techniques.		Removed
Carry out active listening avoiding barriers and using silence/listening to encourage expressing feelings, thoughts and concerns.		Removed
Support the patient in making decisions by informing the patient about the existence of alternative points of view and solutions clearly and with full support.		Removed
Provide the information requested by the patient respecting the patient's right to receive or not receive information.		Removed
Serve as a connection between the patient and family and with other health professionals.		Removed
Identify the situations in which the person being cared for may require spiritual support, implementing actions, within the care plan, to provide spiritual support to the person being cared for.		Removed
Provide an environment that promotes private conversations between the patient, family and health professionals.		Removed
Implement actions to alleviate suffering.		Removed
Identify the situation of the person's grieving process: denial, anger, negotiation, depression and acceptance.		Removed

Support progression through personal stages of grief based on the person's situation.		Removed
**4. Evaluation and quality**		
Monitor the patient's spiritual and emotional situation, through the selected indicators.	Monitor the person’s spiritual and emotional situation, through the selected indicators.	Modified
Monitor the patient's level of suffering through the selected indicators.	Monitor the person's level of suffering through the selected indicators.	Modified
Evaluate the impact of care on the level of patient suffering.	Evaluate the impact of care on the level of suffering of the person.	Modified
Implement improvement actions based on the results, adapting the interventions of the care plan when necessary.	Implement improvement actions based on the results in care of the spiritual and emotional area, adapting the interventions of the care plan when necessary.	Modified
Assess the evolution of the patient's healing process during the care process.		Removed
**5. Communication and interpersonal relationship**		
Show hospitality in welcoming the person, showing interest in their values and expectations.	Show hospitality in welcoming the person, showing interest in their values and expectations.	
Create a climate of intimacy that allows communication on aspects of the spiritual and emotional area of the person.	Create a climate of intimacy that allows communication on aspects of the spiritual and emotional area of the person.	
Identify the situations in which the patient requires spaces of silence and respect them.	Identify the situations in which the person requires spaces of silence and respect them.	Modified
Convey truthfulness, use frank language without hesitation, responding to the patient's doubts.	Transmit truthfulness and use clear language without hesitation, responding to the person's doubts.	Modified
Respect and not judge the ontological dignity of the patient, values and beliefs that may be different from their own.	Do not make judgments and respect the ontological dignity of the person when the values and beliefs are different from their own.	Modified
Plan care considering the moments of intimacy of the patient.	Plan care considering the moments of intimacy of the person.	Modified
	Listen to the expression of feelings about the loss.	New
	Carry out active listening avoiding barriers and using silence/listening to encourage expressing feelings, thoughts and concerns.	New
Respect the confidentiality of the information.		Removed
Offer help to the person cared for to collaborate in the resolution of suffering.		Removed
Implement actions aimed at alleviating suffering.		Removed
Plan care respecting the values and beliefs of the person being cared for and their environment.		Removed
Respect the patient's right not to know.		Removed

**6. Knowledge and intrapersonal development of the student**		
Reflect on one's own vocation, vital values and attitudes, identifying positive and negative attitudes towards the care of the intimate.	Reflect on one's own vocation, vital values and attitudes, identifying positive and negative attitudes towards caring for the spiritual and emotional area.	Modified
Reflect on one's own values and beliefs and identify how they influence caring for others.	Reflect on one's own values and beliefs and identify how they influence caring for others.	
Recognize one's own limits and virtues in spiritual care.	Recognise one's own limits and virtues in spiritual care.	
Recognize the importance of spirituality in your life.	Recognise the importance of spirituality in your life.	
Recognize the signs of emotional, psychic and spiritual exhaustion.	Recognise the signs of spiritual and emotional exhaustion.	Modified
Show personal knowledge by analysing one's own strengths and weaknesses on a spiritual and psychological level.	Show personal knowledge by analysing one's own strengths and weaknesses on a spiritual and emotional level.	Modified
Show self-awareness and emotional control, maintaining self-control in situations of personal suffering.	Show self-awareness and emotional control, maintaining self-control in situations of personal suffering.	
Show a proactive attitude of improvement on a personal level.	Show a proactive attitude of improvement on a personal level.	
Find spaces to stop and connect with yourself: meditation, guided imagery, relaxation.	Find spaces to reflect and connect with yourself: meditation, directed imagination, relaxation.	Modified
Find solutions to the negative influence of one's own values and beliefs in care.	Find solutions to the negative influence of one's own values and beliefs in care.	
Identify situations that cause stress.	Identify situations that cause stress.	
Identify the signs and symptoms of "compassion fatigue" or "cost of caring".	Identify the signs and symptoms of "compassion fatigue" or "cost of caring".	
Relate sensations and experiences in stressful situations to the team.	Relate sensations and experiences in stressful situations to the team.	
Analyse how the patient's situation affects to one's inner life and relationships.	Analyse how affects the situations of the people cared to one's inner life and relationships.	Modified
Respect values and beliefs other than your own.	Respect values and beliefs other than your own.	
Ask for help in situations that you cannot control or resolve.	Ask for help in situations that you cannot control or resolve.	
Learn to treat in a team the intimacy problems identified in the patient.	Learn to treat spiritual and emotional care problems identified in the person as a team.	Modified
